# Concurrent Leydig and Sertoli Cell Tumors Associated with Testicular Mycosis in a Dog: A Case Report and Literature Review

**DOI:** 10.3390/pathogens14080752

**Published:** 2025-07-31

**Authors:** Mirosław Kuberka, Przemysław Prządka, Stanisław Dzimira

**Affiliations:** 1 Private Veterinary Clinic Kuber-Vet, Kazimierza Wlk Str. 3, 63-300 Pleszew, Poland; kubervet@gmail.com; 2 Department and Clinic of Surgery, Faculty of Veterinary Medicine, Wroclaw University of Environmental and Life Sciences, Grunwaldzki Square 51, 50-366 Wroclaw, Poland; przemyslaw.przadka@upwr.edu.pl; 3 Department of Pathology, Faculty of Veterinary Medicine, Wroclaw University of Environmental and Life Sciences, Norwid Str 31, 50-375 Wroclaw, Poland

**Keywords:** mycosis testis, Sertoli cell tumor, interstitial cell tumor, dog

## Abstract

Mycosis is caused by, among other factors, filamentous fungi, ubiquitous molds belonging to *Aspergillus* spp. which are often opportunistic pathogens. Over 100 species of *Aspergillus* have been described. The most common species responsible for diseases in humans and animals are *Aspergillus fumigatus* and *Aspergillus niger*, with *Aspergillus flavus* and *Aspergillus clavatus* being somewhat rarer. *Aspergillus* causes a range of diseases, from localized colonization and hypersensitivity reactions, through chronic necrotizing infections, to rapidly progressing angioinvasion and dissemination, leading to death. Testicular mycosis is extremely rarely described in both humans and animals. No studies in the literature report a simultaneous occurrence of testicular tumors and fungal infection of the organ, so the aim of this paper was to describe, for the first time, a case of two independent testicular tumors coexisting with testicular mycosis. A histopathological examination was performed on the left testicle of a male dog, specifically a mixed-breed dog resembling a husky weighing 22 kg and with an age of 8 years. Bilateral orchidectomy was performed for medical reasons due to the altered outline of the left testicle, leading to scrotal deformation. The dog did not show any clinical signs of illness, and the testicles were not painful. The right testicle, according to the operating veterinarian, showed no macroscopic changes, so histopathological verification was not performed. Microscopic imaging of the changes clearly indicated the coexistence of a tumor process involving Leydig cells (*Leydigoma*, interstitial cell tumor, ICT), Sertoli cells (*Sertolioma*), and fungal infection of the testis. The case suggests the possibility of the coexistence of tumor processes, which may have impaired local immune response of the tissue, with an infectious, in this case fungal, inflammatory process. Based on the literature, this paper is the first report on the occurrence of two independent histotype testicular tumors and their associated mycosis.

## 1. Introduction

Mycosis is caused by, among other factors, filamentous fungi—ubiquitous molds of the genus *Aspergillus* spp.—which are frequently considered opportunistic pathogens. More than 100 *Aspergillus* species have been identified to date, with Aspergillus fumigatus and *Aspergillus niger* being most commonly associated with disease in humans and animals. Less frequently, *Aspergillus flavus* and *Aspergillus clavatus* are implicated. The spectrum of aspergillosis is broad, ranging from local colonization and hypersensitivity reaction to chronic necrotizing infections or rapidly progressing angioinvasion and systemic dissemination, which can result in death.

The incidence of aspergillosis (not to be confused with aspergilloma) has been reported to increase in immunocompromised individuals such as transplant recipients and patients with immunodeficiency syndromes [1,2]. Fungal infection of the upper respiratory tract is the most commonly described form of aspergillosis in both human and veterinary medicine [3,4]. Isolated human cases of aspergillosis involving the urinary bladder and testes have been reported by Martinez Salas et al. and Singer et al. [1,5]. Additionally, Espinoza-Hernandes et al. described testicular sporotrichosis, while Klett et al. reported coccidioidomycosis of the testes and epididymis [6,7]. Focal testicular actinomycosis mimicking a metastatic tumor has also been documented, which indicates a possible similarity of infectious lesions to neoplastic lesions [8]. Furthermore, Petrik et al. reported cutaneous blastomycosis of the scrotum and epididymis, and Totten et al. described blastomycosis involving the prostate and testes in eight dogs [9,10]. Tell conducted an extensive analysis of aspergillosis in both birds and mammals, identifying susceptible species such as dogs, cattle, horses, dolphins, and various domestic avian species [11]. Capilla evaluated the course of aspergillosis across animal species, with the aim of establishing a model applicable to human infection [12]. Venyo reviewed the literature regarding aspergillosis of the urogenital system in both male and female individuals, concluding that testicular aspergillosis is exceptionally rare [13]. The risk of fungal infection is exacerbated by the widespread and often excessive use of antibiotics, as well as by both metabolic and exogenous immunosuppression—including diabetes mellitus, chemotherapy, immunosuppressive therapy, and extensive surgical procedures [13].

Based on the literature, no prior reports have been found describing the simultaneous occurrence of testicular neoplasia and fungal infection of this organ. Therefore, the objective of this study was to present the first documented case of the coexistence of two independent testicular tumor histotypes and aspergillosis affecting the same organ.

## 2. Materials and Methods

Case presentation: A left surgical testis with nodular changes was submitted for histopathological examination from an 8-year-old male mixed-breed husky-type dog weighing 22 kg. Bilateral orchiectomy was performed for medical reasons due to deformation of the scrotum caused by the irregular contour of the left testis. The dog showed no clinical signs of disease, and the testes were not painful on palpation. The dog underwent castration with scrotal ablation under general anesthesia. The wound was closed in layers, and skin sutures were removed on the tenth postoperative day. Postoperative care included analgesics (meloxicam, metamizole) and a five-day course of antibiotics (amoxicillin with clavulanic acid). The wound healed without complications. According to the attending veterinarian, the right testis exhibited no gross pathological changes and was therefore not submitted for histological evaluation.

Material fixed in 10% buffered formalin was sent for histopathological examination. After fixation of the material, it was cut for histopathological processing. The longitudinal section showed an irregularly shaped, well-demarcated, non-encapsulated mass of the testicle, approximately 2 cm in diameter, with numerous irregular cysts of various sizes in the area of the head of the epididymis. These cysts contained a thick, gelatinous fluid with a gray-brown color. Below, a solid, irregular focus of about 1.5 cm in diameter was visible. A strip of the testis, about 1–1.5 cm wide, was visible between these two areas, composed of the thin-walled cysts with brown masses described above. The rest of the testis was solid, with a relatively soft consistency. The material was embedded in paraffin and cut into 3 µm sections. The sections were stained using the routine hematoxylin–eosin (H&E) method. The sections were mounted on Superfrost Plus slides, deparaffinized in xylene, and rehydrated in distilled water. Slides were incubated in Mayer’s hematoxylin for 3 min and then rinsed three times in tap water until the blue coloration stabilized. Subsequently, the sections were counterstained in alcoholic eosin for 10 s without rinsing, followed by dehydration in two changes of 95% ethanol, two of 100% ethanol, and two of acetone. Clearing was performed in two 10 s immersions in xylene. Finally, coverslips were applied to the slides. All evaluations were performed by the same veterinary pathologist. Microscopic examination was carried out using an Olympus BX53 light microscope equipped with an Olympus UC90 camera and cellSens Standard V.1 software (Olympus, Tokyo, Japan).

**Figure 1:**
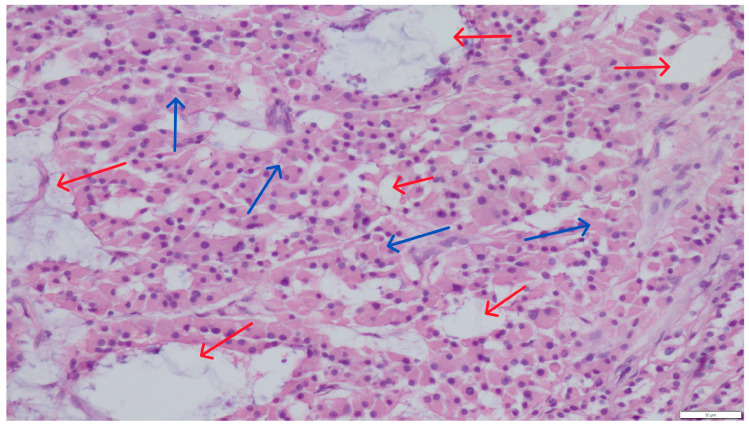
Section of a Leydig cell tumor with a loosely arranged architecture. The neoplastic cells (blue arrows) surround small and large cystic structures (red arrows). Hematoxylin and eosin (H&E) stain (magnification ×200).

Histopathological evaluation of the testicular parenchyma revealed numerous cysts of varying sizes and hemorrhages, surrounded by irregular clusters of interstitial (Leydig) cells. These cells exhibited abundant, mildly eosinophilic cytoplasm and round, slightly eccentrically located nuclei (Figure 1).

**Figure 2:**
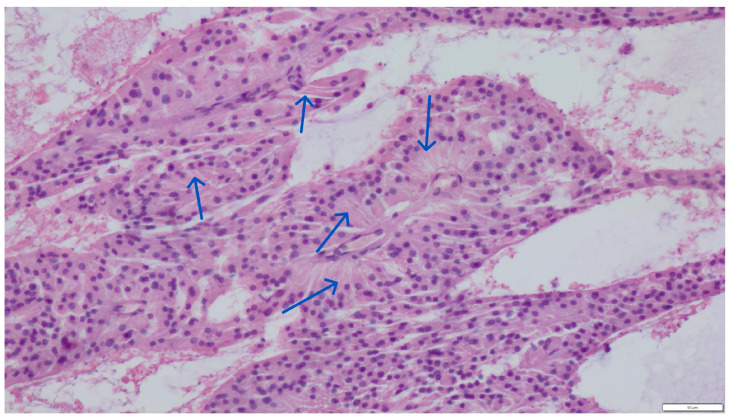
Section of a Sertoli cell tumor, elongated, distinctly cylindrical supporting cells (blue arrows)-diffuse form. Hematoxylin and eosin (H&E) stain (magnification ×200).

Next to these cells’ chaotic, irregular clusters were seminiferous tubules of various sizes, devoid of spermatogonia but with numerous proliferating, elongated, distinctly cylindrical supporting Sertoli cells. The cytoplasm of the Sertoli cells was also mildly eosinophilic. Their round nuclei were located approximately one-third from the base of the cell (Figure 2).

**Figure 3:**
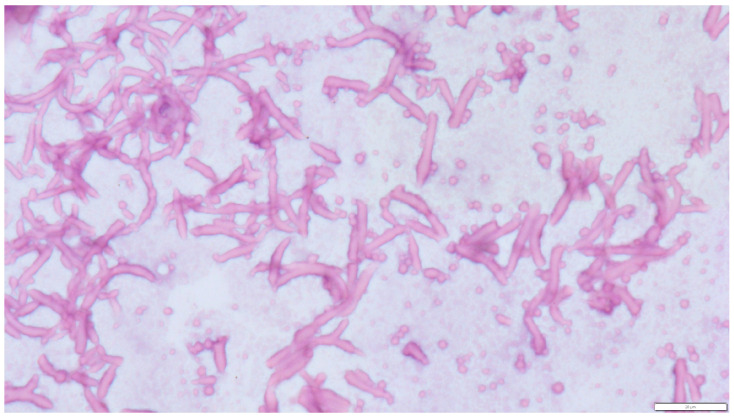
Cystic content showing numerous fragments of septate fungal hyphae. Uniform, septate, and random acute angle dichotomous branching in a progressive arboreal with occasional prominent vesicles was visible. Hematoxylin and eosin (H&E) stain (magnification ×600).

Mitotic activity of both cell types was very low: in the first case (Leydig cells), 0–2 mitoses per field 237 mm 2 (high-power field—HPF, 400×); in the second (Sertoli cells), 0–1 mitoses/HPF. The connective tissue stroma was relatively abundant. The two neoplastic histotypes were located in anatomically distinct regions of the testis and did not exhibit histological intermixing. In the lumen of the cysts were amorphous, weakly acidophilic masses and numerous structures of fungal hyphae consistent with *Aspergillus* spp. Numerous septate hyphae with an acute angle and bulbous dilation were visible. No conidial heads were observed (Figure 3).

**Figure 4:**
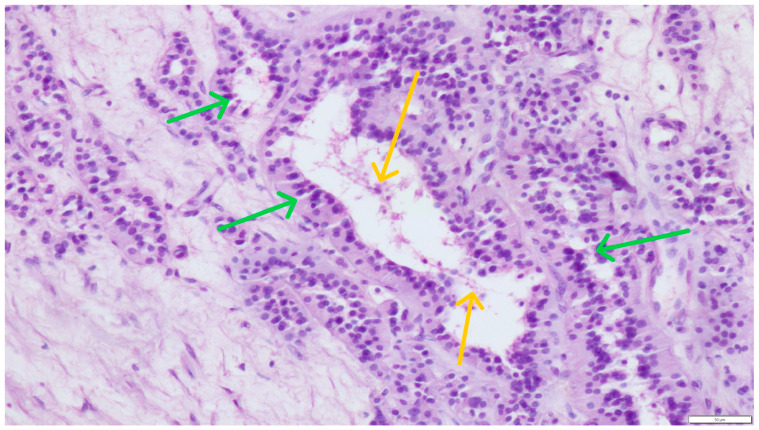
Irregular individual seminiferous tubules with Sertoli cells (green arrows) containing single spermatozoa within the lumen (single sperm heads visible—yellow arrows). Hematoxylin and eosin (H&E) stain (magnification ×200).

On the periphery of the described changes, which dominated in the microscopic image, single, preserved seminiferous tubules with supporting Sertoli cells (green arrows) and single spermatozoa were visible in the lumen (yellow arrows) (Figure 4).

**Figure 5:**
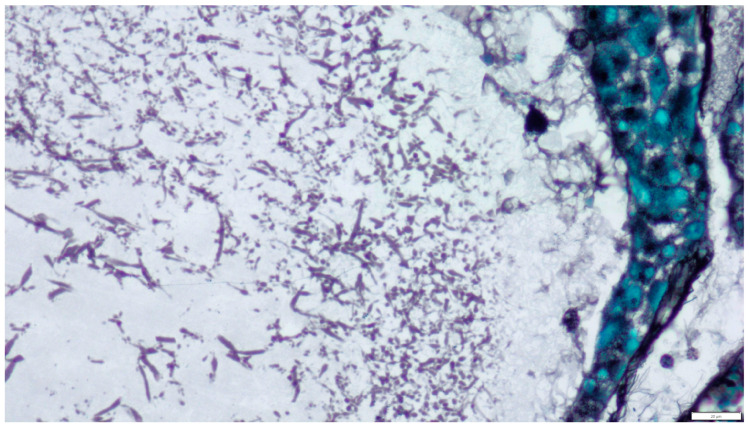
Grocott methenamine silver (GMS) stain demonstrating septate hyphae with acute angle branching in the lumen of the cyst (magnification ×400).

To confirm the presence of fungal structures observed on routine HE staining, Grocott staining was used. This stain highlights the fungal cell walls by staining them black and the surrounding tissues light green (Figure 5).

## 3. Discussion and Conclusions

Mycotic orchitis—caused by, e.g., Aspergillus spp.—in humans has been reported primarily in immunocompromised patients, including organ transplant recipients and individuals infected with human immunodeficiency virus (HIV) [1,2]. The development of fungal infections is facilitated by long-term antibiotic therapy, corticosteroid use, neoplastic disease, and disruption of natural mucosal barriers [1]. Other predisposing factors include metabolic diseases and conditions related to prolonged disturbances in host homeostasis, such as diabetes mellitus, tuberculosis, uropathy, liver cirrhosis, and immunodeficiency syndromes [1]. Among fungal infections of the genitourinary tract, the kidney is the most frequently involved organ, while involvement of other organs is rare [14]. Considering previously published isolated case reports, the presence of *Aspergillus* spp. within the testes appears to be exceptionally rare. Hematogenous dissemination from a primary pulmonary focus is suspected to be the main route of infection. However, an ascending infection from the urethra, reaching the epididymis and testis, is also considered possible [1]. Siemieniuch et al. (2009) described a case of testicular aspergillosis, considering the ejaculate, although the dog failed to become a successful stud [15]. Walker et al. (2012) reported a case of a bitch with pyometra following mating, in which disseminated aspergillosis affecting the uterus and bones was diagnosed [16]. In the present case, due to the lack of clinical history, it was not possible to determine the exact route of testicular infection of the reproductive organs. However, as the dog showed no clinical signs—particularly from the respiratory or cardiovascular systems—and was considered fit for elective surgery, it is unlikely that the respiratory tract served as the primary site of infection. As noted previously, neoplastic processes and local immunosuppression may be suggested to act as predisposing factors for opportunistic fungal infections. Pathogenic fungi, e.g., *Aspergillus* spp., are ubiquitous opportunistic pathogens [3]. Thus, infection may have been initiated through colonization of the penis, followed by ascending spread to the testis and epididymis. Although percutaneous infection through the preputial or scrotal skin is possible, the referring clinician did not report any wounds in this region or the need for scrotectomy. In our case, the identification of *Aspergillus* spp. has not been molecularly confirmed.

Leydig cell tumors and Sertoli cell tumors, along with seminomas, are among the most frequently diagnosed testicular neoplasms in dogs. These tumors typically occur unilaterally, although multiple unilateral or bilateral presentations are also observed, often accompanied by hemorrhage or foci of necrosis. Malignant forms of these tumors are rare (e.g., Sertoli cell tumor) or extremely rare (e.g., Leydig cell tumor). The occurrence of histologically distinct testicular tumors within the same testis has been reported in both humans [17,18,19,20,21,22] and animals, including dogs [23,24,25,26,27] and a stallion [28]. Manuali et al. (2020), in a retrospective study of 388 canine testicular tumors in central Italy (Umbria), found that 10% of cases exhibited multiple tumor types within a single testis [26]. The most common combination was seminoma and Leydig cell tumor (SEM–ICT; 14 cases, 63.6%), followed by SEM–SCT (4 cases, 18.2%). Only two dogs had ICT–SCT (9.1%) and ICT–SEM–SCT combinations (9.1%). In another study by Grieco et al. (2008), involving 232 dogs, 19 cases (8.18%) showed multiple tumors within a single testis [24]. The most common combination was again SEM–ICT (8 cases, 42%), followed by ICT–SCT (5 cases, 26%), SEM–SCT (4 cases, 21%), and SCT–ICT–SEM (2 cases, 11%). A study by a Brazilian research group revealed that out of 190 dogs with testicular neoplasms, 30 (15.8%) had more than one tumor type. Sixteen dogs (8.42%) had multiple neoplasms confined to one testis, while in four cases, different tumors affected both testes. The most common combinations were SEM–ICT (16 dogs), SEM–SCT (12 dogs), and SCT–ICT (2 dogs) [25]. Liao et al. (2009) reported the occurrence of multiple and different testicular neoplasms in 16.8% (80/476) of dogs studied [29]. Of these, unilateral multiple tumors were diagnosed in 22 dogs (4.62%). Among them, the most frequent combination was SCT–SEM (12 cases), followed by SEM–ICT (5 cases) and SCT–ICT (4 cases). The highest proportion of multiple tumors in a single testis was observed by Slovenian researchers, who examined 206 dogs and documented 301 testicular tumors [27]. Of these, 37 cases (18%) featured two or more histologically distinct tumor types localized unilaterally. The combinations were SEM–ICT (12 cases, 32.4%), SEM–SCT (9 cases, 24.3%), SCT–ICT (2 cases, 5.4%), and SEM–SCT–ICT (2 cases, 5.4%). The case presented here illustrates the possibility of the coexistence of multiple and different testicular neoplasms, potentially resulting in significantly reduced local immunity and predisposing the tissue to secondary infectious processes such as fungal inflammation.

To the authors’ knowledge, only a few reports describing infection of the canine reproductive tract with fungi have been previously published. Siemieniuch et al. [15] described a testicular *Aspergillus* spp. infection in a breeding dog and transmission of the infection to two bitches during mating. Walker et al. [16] described a bitch with pyometra due to *Aspergillus* spp. Other fungal reproductive tract infections are blastomycoses described by Totten et al. [10]. Diagnosis of mycoses can be performed in several stages (phases):

– Clinical analysis and laboratory test results: Morphological, biochemical, serological, and other;
– Collection of various biological materials from patients for mycological evaluation: Blood, cerebrospinal fluid, and fluids from the pleural cavity, peritoneum, and joints; material from cysts, abscesses, and puncture of the paranasal sinuses; samples from the lower respiratory tract (e.g., sputum); samples obtained during surgical procedures, endoscopy, or fine-needle aspiration biopsy; swabs from the oral cavity, pharynx, nasal cavities, and larynx; content from the genital organs, for example, from the urethra, urine, and others;
– Direct techniques are the simplest diagnostic method, and sometimes, they are decisive, such as in *Pneumocystis carinii* or *Rhinosporidium seeberi* infections;
– Stained preparations: smears, histopathological preparations;
– Cultures—Material collected for testing for the presence of fungi is inoculated onto liquid or solid media—containing protein, carbohydrates, and other components—directly in the amount of 0.5–1.5 cm^3^. Tissues or biopsy specimens are homogenized or ground by shaking with sterile beads; materials collected during biopsy are simultaneously inoculated onto solid or liquid Sabouraud media or broth agar;
– Other techniques include fluorescence, immunohistochemistry, immunofluorescence, and in situ hybridization. Immunohistochemical tests with monoclonal antibodies (WF-AF-1, EB-A1), immunofluorescence, and in situ hybridization are also used in diagnostics to determine the genus and species, especially in cases where it is not possible to culture fungi [3,13,14,16].

This case is consistent with the conclusions of Petrik et al. [9], which emphasize the importance of considering endemic fungal infections in the differential diagnosis of epididymo-orchitis. Urologists often focus on bacterial and viral infections, but fungal and mycobacterial infections should remain on our list of differentials. Tissue culture for bacteria, fungi, and mycobacteria is essential for diagnosing such pathogens and greatly accelerates treatment.

Unfortunately, a lack of precise data about the patient and the impossibility of assessing the entire reproductive system prevent the determination of the route and mechanism of fungal infection. Due to the lack of unfixed tissue and the material being formalin-fixed paraffin-embedded (FFPE), additional molecular diagnostics such as PCR or culture were not feasible. Therefore, the identification of fungal elements such as *Aspergillus* spp. remains presumptive and is based solely on morphological features and GMS staining.

Despite these limitations, this is, based on the literature, the first documented case describing the simultaneous presence of two distinct testicular neoplasms and testicular aspergillosis mycosis (probably aspergillosis) in a dog.

## Data Availability

Data are contained within the article.

## Abbreviations

HE	Hematoxylin and eosin (stain)
HIV	Human immunodeficiency virus
HPF	High-power field
ICT	Interstitial cell tumor
SCT	Sertoli cell tumor
SEM	Seminoma
